# When a Lower Abdominal Lump Turned Out to Be a Stromal Tumor of Unknown Malignant Potential

**DOI:** 10.7759/cureus.98221

**Published:** 2025-12-01

**Authors:** Shivika Jindal, Ganesh Guru

**Affiliations:** 1 General Surgery, Sree Balaji Medical College and Hospital, Chennai, IND

**Keywords:** abdominopelvic mass, malignancy, stromal tumor of unknown malignant potential (stump), tumor, uterine smooth muscle tumor

## Abstract

Abdominopelvic masses usually do not occur in young females and should be evaluated carefully to rule out rare smooth muscle tumors. Here, we present the case of a 23-year-old female who complained of pain in the lower abdomen, urinary frequency, constipation, and dysuria for the last two months. The examination showed a solid, immobile lower abdominal mass measuring approximately 20 × 25 cm. MRI revealed a clear-cut abdominopelvic lesion measuring 7.9 × 19.7 × 30.4 cm, and fluorodeoxyglucose-positron emission tomography depicted subtle hypermetabolic activity, indicative of a low-grade stromal lesion. During an exploratory laparotomy, a large mass arising on the right broad ligament was noted, and total excision was performed. Histopathology revealed a smooth muscle tumor with mild-to-moderate atypia, low mitotic activity, and absence of coagulative necrosis, which was consistent with a diagnosis of stromal tumor of unknown malignant potential (STUMP). The course after surgery was uneventful, and at the six-month follow-up, the patient did not show any metastasis or recurrence. This case described the diagnostic difficulties of extrauterine STUMP and the significance of histopathological verification and regular follow-up surveillance.

## Introduction

Gynecological pelvic masses in women of reproductive age need early investigation to determine the benign processes and rare tumor occurrences [1,2]. Uterine smooth muscle tumors can be seen in women aged between 30 and 50 years, with benign leiomyoma being the most common. A minor group of tumors has atypical histological characteristics and is known as smooth muscle tumor of uncertain malignant potential (STUMP) [3]. Recurrence rates are reported to range approximately 7-20% with infrequent development of leiomyosarcoma during follow-up, highlighting the need for long-term monitoring [3-6]. The preoperative diagnosis is also troublesome, as ultrasonography and MRI usually present vague appearances and can be similar to leiomyoma [3]. Hence, the practice is based on imaging as an indicator of the extent of the tumor and the involvement of the organs, and the conclusive diagnosis is confirmed with the help of postoperative histopathological examination. A tumor is a STUMP when it does not meet certain parameters to diagnose leiomyosarcoma, namely, in terms of mitotic rate, cell atypia, and the absence or presence of coagulative tumor cell necrosis (CTCN) [7]. No standard management guidelines apply to STUMP, but complete surgical excision is usually advised, with hysterectomy being the preferred option in patients who have already given birth and myomectomy in younger women who desire to preserve fertility [5,6]. This case of a broad ligament STUMP in an unusual location in a young female at the age of 23 years presents diagnostic uncertainties, pointing to the need to consider surgical decision-making in young individuals and the significance of organized postoperative monitoring.

## Case presentation

**Figure 1:**
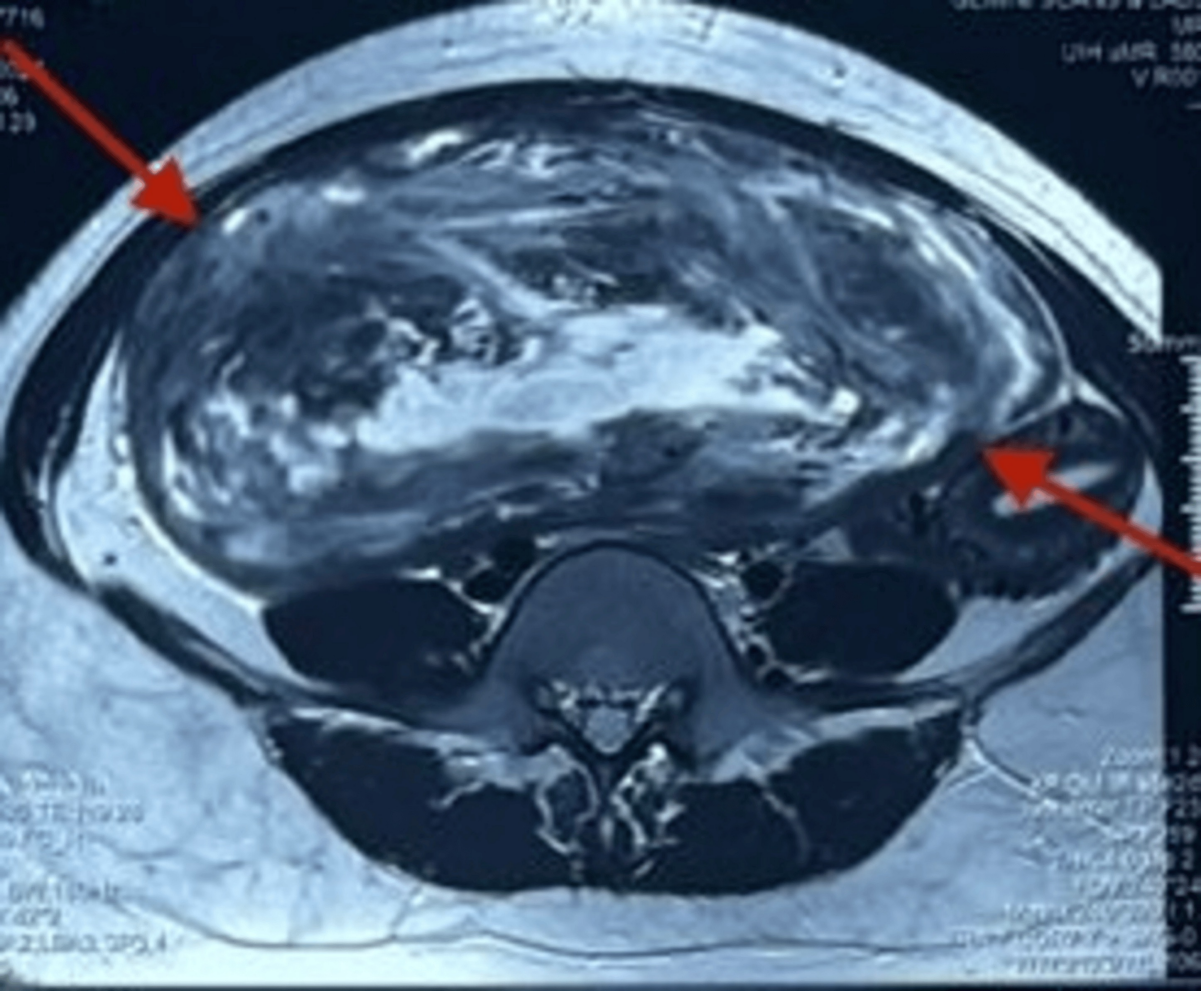
MRI Abdomen revealed a large relatively well defined abdomino-pelvic non diffusion restricting T2 heterointense lesion with central T2 hyperintensity in the mesentric plane measuring 7.9x19.7x30.4 cm (MRI: Magnetic Resonance Imaging)

A 23-year-old unmarried female presented with a history of intermittent, dull, non-radiating lower abdominal pain that started about two months ago. She had also experienced dysuria and increased urinary frequency during the past 15 days. She also complained of constipation and the free passage of flatus. Other symptoms were difficulty in breathing and a lack of appetite for one week. She experienced irregular menstrual cycles and had no significant surgical, medical, or family history. On examination, the patient was afebrile and hemodynamically stable. Inspection of the abdomen revealed fullness of the lower abdomen. Palpation revealed a firm-to-hard, immobile mass with poorly defined margins measuring 20 × 25 cm and located in the hypogastric and right iliac areas. Bowel sounds were sluggish. Digital rectal examination was normal. Laboratory tests, such as complete blood count and renal function tests, were normal. Initially, a pelvic ultrasonography was performed, but the size and extrauterine nature of the lesion restricted the use of sonographic characterization. Typically, uterine leiomyomas demonstrate well-circumscribed, hypoechoic masses, but atypical features, such as heterogeneous echotexture, cystic degeneration, or unclear uterine attachment, may raise suspicion for alternative pathology. MRI of the abdomen (Figure 1) revealed a 7.9 × 19.7 × 30.4 cm well-defined abdominopelvic mass with T2 heterointensity and central hyperintensity located in the mesenteric plane, without diffusion restriction.

**Figure 2:**
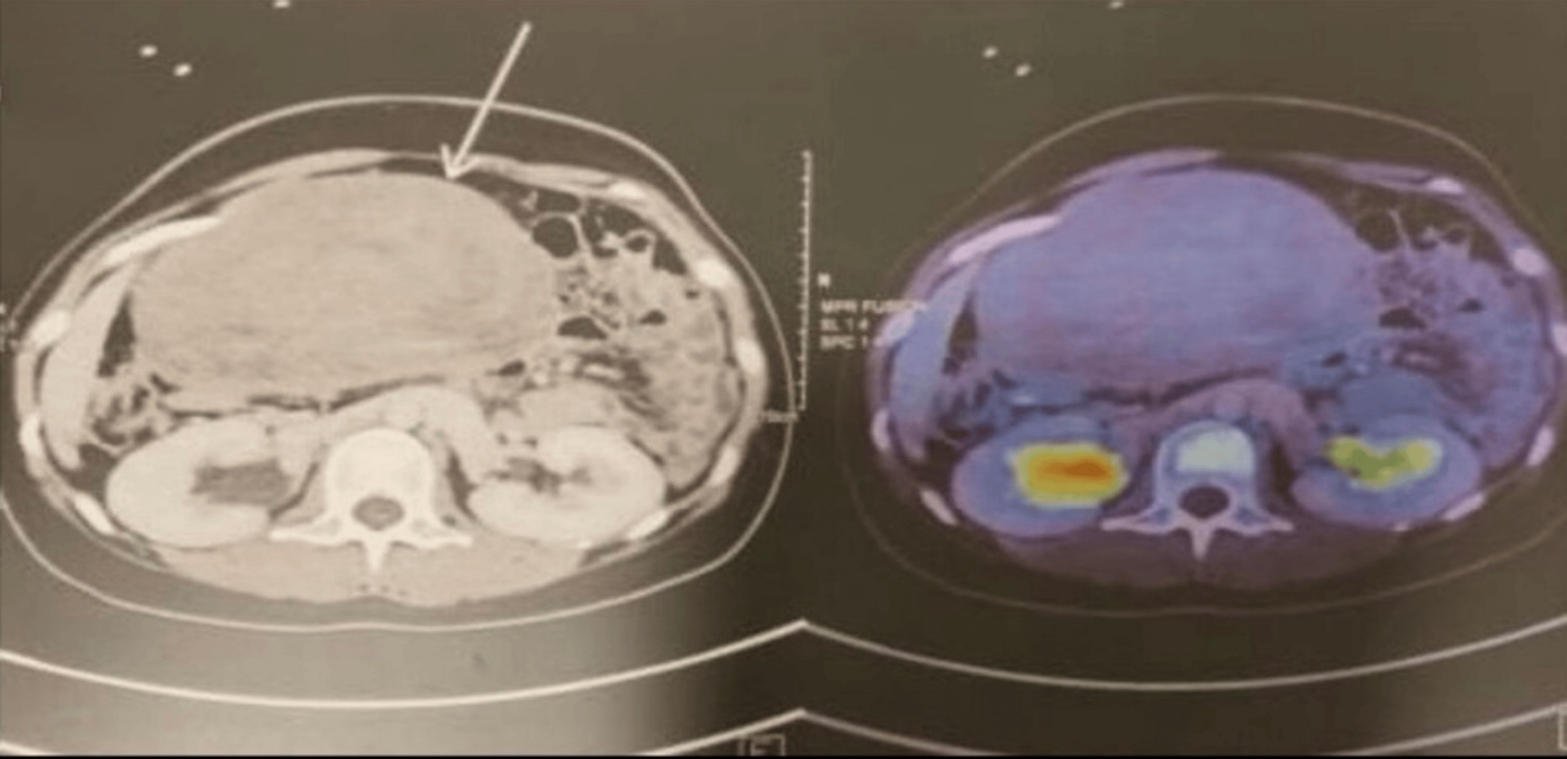
Fluorodeoxyglucose-positron emission tomography scan showing a faintly hypermetabolic, relatively well-defined, lobulated lesion in the abdominopelvic region measuring 199 × 83 × 295 mm, suggesting a low-grade stromal tumor of right adnexal origin.

Whole-body fluorodeoxyglucose-positron emission tomography (Figure 2) imaging demonstrated a faintly hypermetabolic, lobulated mass measuring 199 × 83 × 295 mm, suggesting a low-grade stromal tumor, initially suspected to arise from the right adnexa.

**Figure 3:**
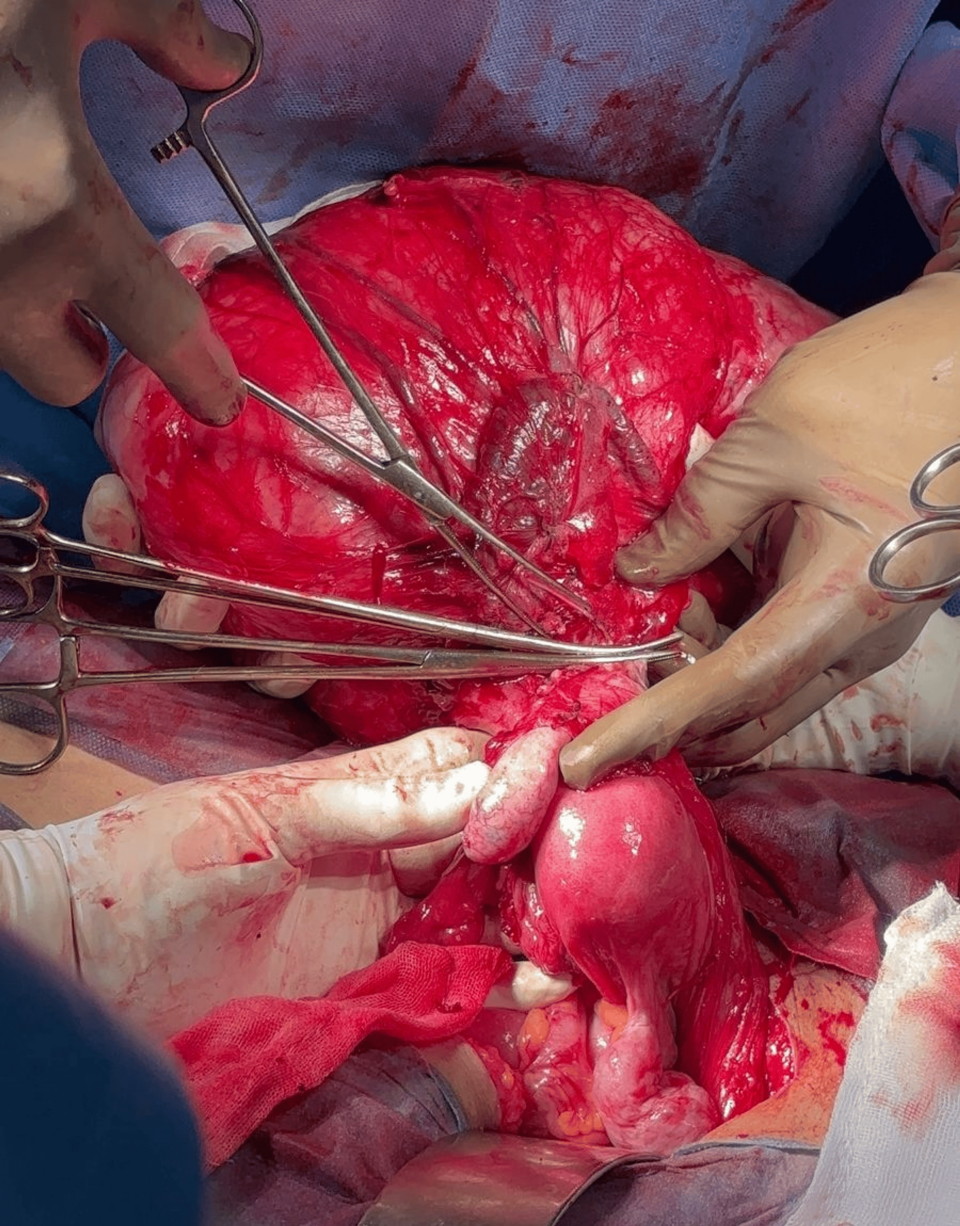
Surgical resection of the abdominopelvic mass arising from the right broad ligament, separate from the uterus and adnexa.

**Figure 4:**
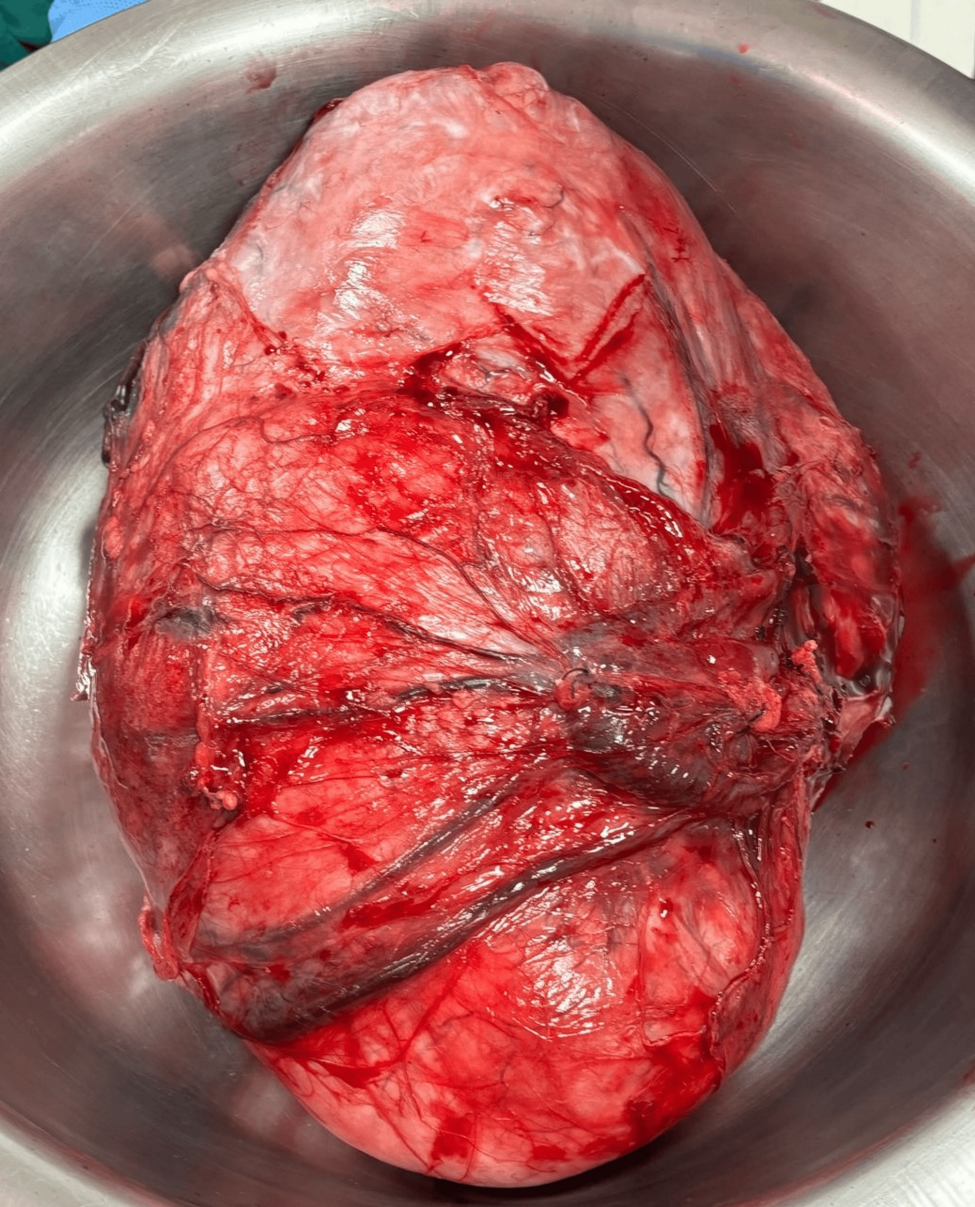
Resected abdominopelvic mass arising from right broad ligament weighing 1.5 kg.

The patient underwent surgery in February 2025 in Chennai. Intraoperatively, the mass was found arising from the right broad ligament, separate from the uterus and adnexa, confirming its extrauterine origin (Figure 3). Complete excision was achieved without rupture, and the mass weighed 1.5 kg (Figure 4).

**Figure 5:**
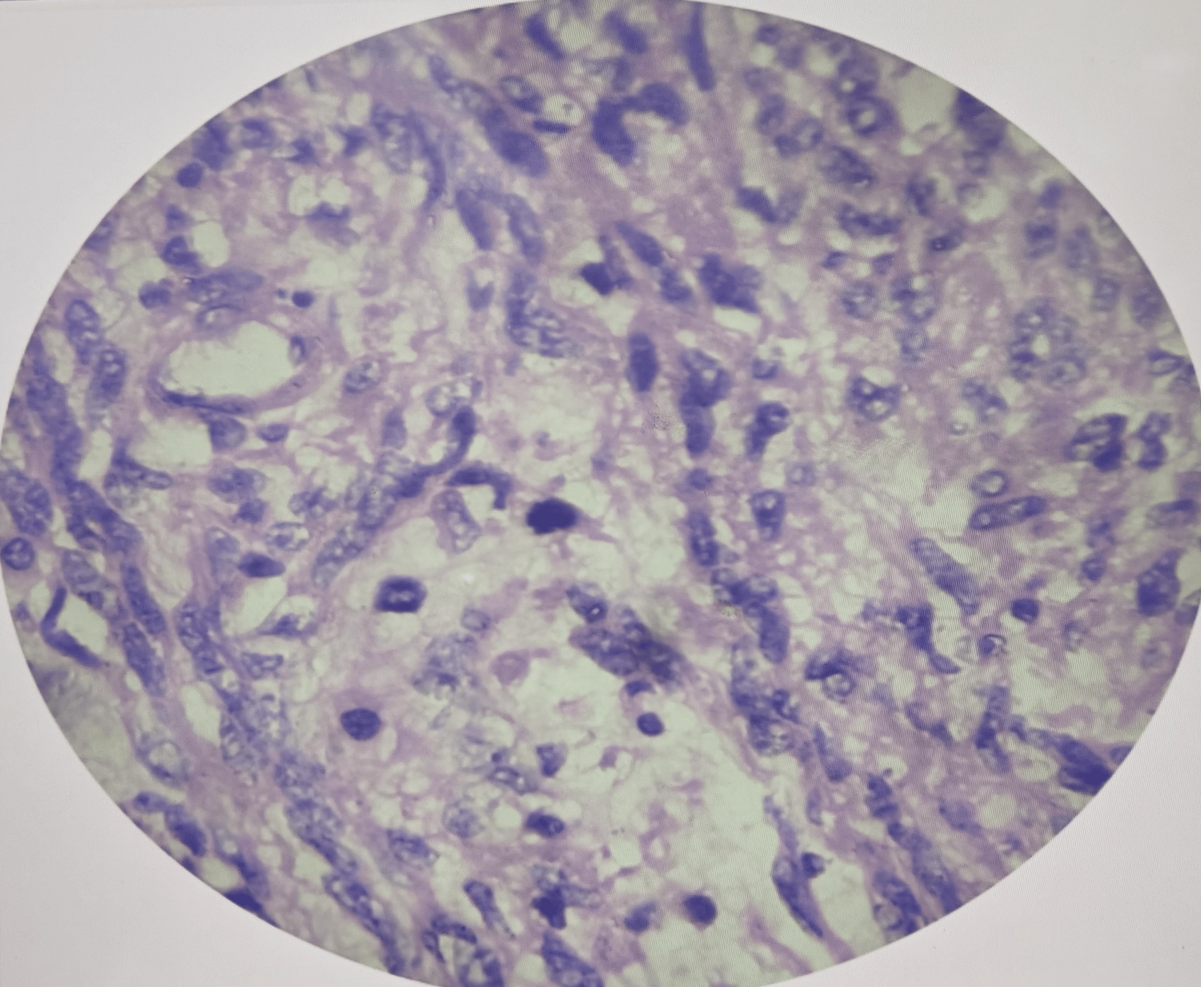
Histopathological examination showing mild-to-moderate cytologic atypia and low mitotic activity and no coagulative tumor cell necrosis.

Gross pathological examination revealed a well-circumscribed smooth muscle tumor with focal areas of increased cellularity. Microscopic evaluation demonstrated mild-to-moderate cytologic atypia, low mitotic activity (3-5 mitoses per 10 high-power fields), and absence of coagulative tumor cell necrosis (Figure 5).

These findings did not meet the criteria for leiomyosarcoma but were not consistent with a benign leiomyoma, supporting a diagnosis of STUMP. Immunohistochemistry, when performed, typically aids risk stratification; however, in this case, it was not performed as the patient did not consent to additional testing. The postoperative course was uneventful, and the patient was discharged on postoperative day seven. Complete surgical excision was selected due to the size of the mass, progressive symptoms, and uncertain origin, as tissue diagnosis could not be obtained safely through less invasive means. Myomectomy was feasible because the tumor arose from the broad ligament and did not involve the uterus, allowing fertility preservation. While hysterectomy is considered in women who have completed childbearing, individualized decision-making remains essential, particularly in young patients. In this case, organ-preserving surgery was appropriate, and no recurrence was detected at the sixth-month follow-up. The patient remains under ongoing surveillance due to the unpredictable behavior associated with STUMP.

## Discussion

STUMPs are uncommon neoplasms whose clinical course is erratic and are frequently only diagnosed once they are removed surgically [3]. In this case, the lesion had its origin in the broad ligament, which is an unusual location, as most STUMPs are noted in the uterus. In this case, the pressure-related symptoms were due to the large tumor size, and other stromal tumors could not be reliably differentiated by imaging. Due to their similarities with benign leiomyoma in clinical and radiographic appearance, preoperative differentiation is challenging despite the presence of an enlarged pelvic mass and pressure effects on other structures [3]. The diagnostic uncertainty is also enhanced by an insufficient knowledge of the prognostic factors and biological behavior [5]. The classification still relies on histopathological examination, which is based on three main parameters, namely, mitotic activity, cytologic atypia, and CTCN [6]. The tumor in the present case had a mild-to-moderate atypia, low mitotic index (3-5/10 HPF), and no CTCN, indicating that it was a STUMP and not leiomyosarcoma. Such borderline characteristics can be used to understand why the clinical course is unpredictable and why imaging or intraoperative impression should not be used on its own.

As STUMPs are uncommon, the literature on the subject is limited, and there are no unified guidelines regarding optimal management or monitoring [3]. Surgical excision is usually considered the optimal treatment modality, but the treatment should be personalized. Hysterectomy is generally recommended to women who have had a pregnancy, whereas fertility-sparing procedures can be a suitable option in younger patients as long as they are advised about the possibility of recurrence [6]. Although in the majority of patients the disease does not recur, it has been reported [6]. In case of hormonal responsiveness, another possible alternative in fertility-preserving approaches can be the levonorgestrel-releasing intrauterine system [7]. The understanding of STUMP as a diagnostic possibility in patients with atypical or rapidly enlarging uterine or extrauterine smooth muscle masses can help make the correct choice promptly and to use postoperative monitoring as a well-structured procedure, especially in young patients, where organ preservation plays a significant role [8]. According to recent research, adjuvant options such as progesterone therapy, gonadotropin-releasing hormone analogs, or chemotherapy did not prove to be regularly beneficial in preventing recurrence [9,10]. Follow-up intervals are also not agreeable. According to Ip et al., the clinical and imaging surveillance should be performed every six months for the first 5 years, followed by annual monitoring for an additional five years [10].

## Conclusions

We presented an unusual case of broad ligament STUMP in a young woman, demonstrating the challenge associated with preoperative diagnosis. The imaging methods are not able to reliably differentiate between STUMP and other smooth muscle tumors, making histopathological confirmation crucial. Most cases are benign; however, the possibilities of recurrence necessitate long-term follow-up and proper counseling of the patient. This case supports the idea of individualized treatment and further awareness of STUMP as an uncommon yet significant differential diagnosis of large pelvic smooth muscle tumours.
